# Right atrial myxoma after Amplatzer atrial septal defect closure device deployment

**DOI:** 10.1093/icvts/ivad025

**Published:** 2023-02-03

**Authors:** Pan Li, Laichun Song, Liang Tao, Xufa Chen

**Affiliations:** Department of Cardiac Surgery, Wuhan Asia Heart Hospital, Wuhan, China; Department of Cardiac Surgery, Wuhan Asia Heart Hospital, Wuhan, China; Department of Cardiac Surgery, Wuhan Asia Heart Hospital, Wuhan, China; Department of Cardiac Surgery, Wuhan Asia Heart Hospital, Wuhan, China

**Keywords:** Right atrial myxoma, Amplatzer atrial septal defect closure device

## Abstract

Right atrial (RA) myxoma is common; however, RA myxoma after the percutaneous atrial septal defect closure is very rare. To the best of our knowledge, this might be the first reported case of RA myxoma after Amplatzer closure of an atrial septal defect, leading to a pulmonary artery embolism. We removed all the RA mass, occluder and pulmonary embolus and reconstructed the atrial septum successfully. As a result of surgery, there were no other complications as following up.

## INTRODUCTION

Acute pulmonary embolism represents a severe condition associated with significant morbidity and mortality. The main source of pulmonary embolism is represented by deep venous thrombosis. Less frequent causes are right-sided heart chamber clots, neoplastic emboli into the systemic veins or the association of right atrial (RA) thrombi and atrial septal defects (ASDs). We describe a unique case of acute pulmonary embolism related to the RA myxoma after Amplatzer ASD closure device deployment.

## CASE REPORT

A 72-year-old woman after percutaneous secundum ASD closure 4 years ago was transferred to our clinic with exertional dyspnoea and haemoptysis. Because of hypoxaemia and inverted T waves in the right precordial leads, pulmonary thrombo-embolus was suspected.

**Figure 1:**
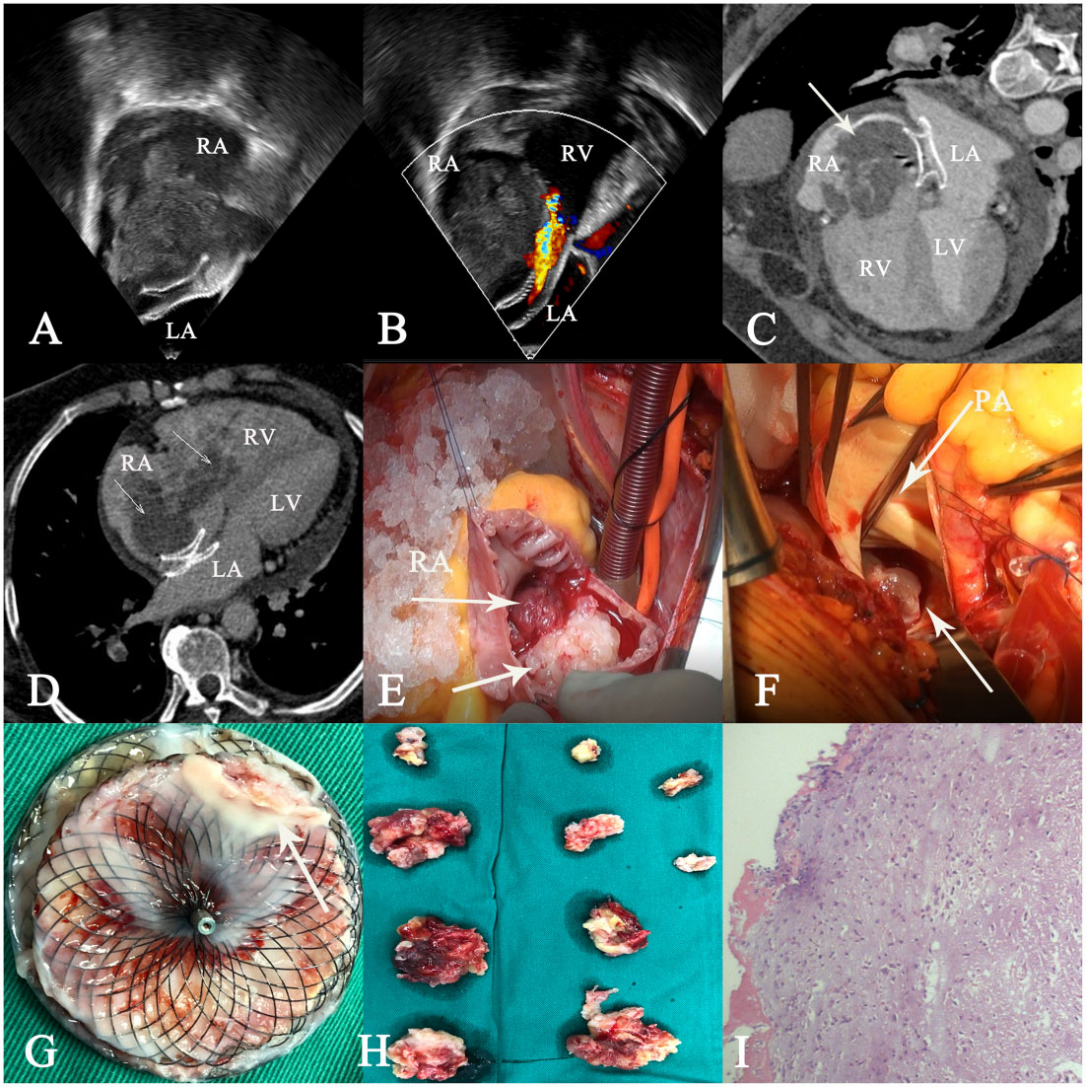
(**A** and **B**) Transesophageal echocardiography confirmed the presence of an irregular and inhomogeneous mass attached to the occluder’s superior edge, without obvious pedicle. (**C** and **D**) Computed tomography angiography showed a large mass in the right atrium and ventricular attaching the occluder edge and more embolic fragment occluding the pulmonary segment artery. (**E**–**G**) The right atrial mass, occluder and pulmonary embolus were removed; reconstruction of the atrial septum was done. (**H**) Myxoma removed from the right atrium and pulmonary artery. (**I**) The right atrial myxoma and pulmonary embolus with tumour were confirmed by histopathological study.

Transthoracic echocardiography revealed a moving structure (74 mm × 34 mm) in right atrium, slightly flopping into right ventricular inflow tract with moderate tricuspid regurgitation. Transoesophageal echocardiography confirmed the presence of an irregular and inhomogeneous mass attached to the occluder’s superior edge, without obvious pedicle (Fig. 1 A and B). Computed tomography angiography showed a large mass in the right atrium cling to the occluder edge and more embolic fragment occluding the pulmonary segment artery (Fig. 1 C and D). The operation was performed under a standard cardiopulmonary bypass. The RA mass, occluder and pulmonary embolus were removed; reconstruction of the atrial septum was done (Fig. 1 E–G). Surgical excision showed much brownish, irregularly shaped, gelatinous mass attached to the device (Fig. 1 I). The main pulmonary artery was dissected longitudinally, which was filled with tumour thrombus, and the pulmonary embolus was completely removed, and the proximal and distal parts of the main pulmonary artery were explored without obvious residual pulmonary embolus (Fig. 1). Then, pathological examination confirmed myxoma, as did the RA mass. The RA myxoma and pulmonary embolus with tumour were confirmed by the histopathological study. Patient was discharged in good condition remaining under follow-up of cardiology outpatients.

## COMMENT

Presently, we believe that there is only 1 report in the English literature that describe RA myxoma complications after surgical correction of ASD, in which the patient presented with hypertension. Suzuki *et al.* [1] described a 13-year-old patient with hypertension ∼4 years after primary surgical repair of ASD. In this case, the myxoma arose from the atrial septum in the area between the orifices of the coronary sinus and inferior vena cava without involving the suture line. Several cases of left atrial myxoma have been described after percutaneous ASD closure, in which the patients presented without symptoms and underwent successful surgical resection [2, 3]. The present case is unique in that the elderly patient with a large RA myxoma after percutaneous ASD closure complicated with pulmonary artery obstruction underwent successful surgical correction. To our knowledge, this might be the first reported case of RA myxoma after Amplatzer closure of an ASD.

The case highlights the importance that attention should be paid to the possibility of myxoma after percutaneous ASD closure. The growth rate in this case was indistinct for breaking off to pulmonary embolism, suggesting that growth rate alone is not an adequate distinguishing feature for thrombus versus myxoma. Furthermore, the workup revealed limitations of current imaging modalities to discern between organized clot and atrial myxoma. CT might have provided more detailed information, which was performed given the suspicion for thrombus and pulmonary artery obstruction. Due to the patient's previous percutaneous ASD closure in another hospital, and after 4 years, the imaging data before percutaneous procedure were lost to follow up.

Because of the rarity of such cases, although we cannot be certain that the occluder was involved in the formation of the RA myxoma in this case, the pedicled myxoma was closely associated with the occluder on the basis of the intraoperative findings. In this case, pulmonary embolism could have been associated with the large mass, and the pathological examination confirmed myxoma, as did the RA mass. This case may indicate a causal relationship between the ASD occluder and the myxoma, and the development of the myxoma is considered one of the possible complications after ASD closure with occluder device. Currently, the histogenesis of myxoma is poorly understood; however, the mesenchymal and endothelial properties of myxoma cells suggest that a clearer understanding of tumour origins can be achieved through a detailed investigation of heart development and endocardial histogenesis. Further in-depth studies are needed to prove these explanations.

In conclusion, RA myxoma at the site of percutaneous ASD closure is an uncommon complication that has the potential for embolic sequelae. The present case demonstrates the importance of postoperative interval imaging to detect potential thrombi or myxoma prior to manifestation by embolic event.
